# Associations between adherence to MIND diet and metabolic syndrome and general and abdominal obesity: a cross-sectional study

**DOI:** 10.1186/s13098-020-00611-6

**Published:** 2020-11-18

**Authors:** Saba Mohammadpour, Parivash Ghorbaninejad, Nasim Janbozorgi, Sakineh Shab-Bidar

**Affiliations:** Department of Community Nutrition, School of Nutritional Sciences and Dietetics, Tehran University of Medical Sciences (TUMS), No 44, Hojjat-dost Alley, Naderi St., Keshavarz Blvd, Tehran, Iran

**Keywords:** MIND diet, Metabolic syndrome, General obesity, Central obesity, Cross-sectional

## Abstract

**Background:**

There is a lack of studies examining the association between Mediterranean-DASH Intervention for Neurodegenerative Delay (MIND) and metabolic syndrome (MetS) and obesity. Thus, this study aimed to investigate the association of adherence to the MIND diet with MetS and general and abdominal obesity.

**Methods:**

This cross-sectional study was performed on 836 Iranian adults, 18–75 years old. A 167-item food frequency questionnaire (FFQ) was used to assess dietary intakes of participants. Anthropometric measurements, blood pressure, fasting blood glucose and lipid profile of each participant were recorded. The guidelines of the National Cholesterol Education Program Adult Treatment Panel III (ATP III) was used to define MetS.

**Results:**

Mean age of study participants was 47.7 ± 10.7 years. The prevalence of MetS was 36.1% and mean body mass index (BMI) and waist circumference (WC) was 27.7 ± 4.69 kg/m^2^ and 92.0 ± 12.4 cm respectively. Those who were in the third tertile of the MIND diet score compared to the first tertile had 12% lower odds of having the MetS (ORs: 0.88; 95% CI 0.62–1.24) but the association was not significant (P = 0.77). There was a significant inverse association between the MIND diet score and odds of reduced high-density lipoprotein cholesterol (HDL-C) (ORs: 0.59; 95% CI 0.41–0.85; P = 0.008) and general obesity (ORs: 1.190.80–1.78; 95% CI 0.80–1.78; P = 0.02) in crude model and after controlling for confounders.

**Conclusions:**

We found that the MIND diet score is inversely associated with odds of reduced HDL and general obesity in Iranian adults.

## Background

Metabolic syndrome (MetS), is a collection of metabolic disorders that acts as a risk factor for cardiovascular diseases (CVD) and type 2 diabetes mellitus (T2D) [1]. People with three or more symptoms including high fasting blood sugar (FBS), hypertriglyceridemia, high blood pressure, low high-density lipoprotein cholesterol (HDL-C), and abdominal obesity are identified as MetS according to the National Cholesterol Education Program (NCEP) Adult Treatment Panel-III (ATP III) [2]. Obesity is one of the most major factors contributing to a high prevalence of MetS and other diseases [3]. The prevalence of MetS continues to rise with increasing obesity rates worldwide [4, 5].

Nutritional factors are among the most important factors implicated in the etiology of MetS and obesity. Given the fact that people do not receive nutrients individually and receive nutrients in a context of diet, some studies have examined the effects of dietary patterns such as Mediterranean dietary pattern (MD) and Dietary Approaches to Stop Hypertension (DASH) on MetS but the findings were inconsistent [6–8]. In some studies, adherence to the DASH diet was inversely associated with odds of MetS and some of its components including elevated blood pressure, serum HDL-C, serum triglyceride (TG) and high waist circumference (WC), and body mass index (BMI) in Iranian population [6, 8], but this association was not observed in European patients [7]. Also, some components of the MD have been related to a lower prevalence of MetS criteria and insulin resistance [9]. In addition, a cross-sectional study demonstrated no association between MD and MetS in patients with T2D [10].

Recently, Mediterranean-DASH Intervention for Neurodegenerative Delay (MIND) was identified as a new dietary pattern, which is a combination of Mediterranean-DASH diets [11]. That has been shown to be effective in brain health and many studies have shown the association of the MIND diet and neurological diseases [12, 13]. The MIND diet emphasizes 10 components, that are brain-healthy foods including green leafy vegetables, other vegetables, berries, nuts, beans, whole grains, fish, poultry, olive oil, and wine; and 5 brain-unhealthy foods including cheese, butter or margarine, fast foods or fried foods, red meat and pastries or sweets [13].

Therefore, unlike the DASH and MD diets, it emphasizes on consumption of green leafy vegetables and berries, not other types of vegetables and fruits, and a separate category for cakes and pastries. Also includes fast foods, fried foods, butter and margarine that they had not been included in the DASH or MD diet [13].

There are limited studies on the relationship between the MIND diet and obesity. Aminianfar et al. [14] found no significant association between adherence to the MIND diet and general and central obesity in a sample of Iranian adults live in Isfahan. However, the relationship between the MIND diet and MetS has not been assessed. Due to the continuous increase in MetS prevalence during the last few decades and an increasing rate of obesity, we aimed to investigate the relationship between adherence to the MIND diet and MetS and obesity in Iranian adults.

## Materials and methods

### Study design and participants

This cross-sectional study was performed on 850 Iranian adults (20–59 years old) who referred to Health centers in five regions of Tehran: North, South, East, West, and Central. After the random selection of Health centers, an identical number of subjects were randomly chosen from each center. Individuals who had at least one incomplete variable were excluded and finally, 836 adults remained.

Inclusion criteria were being 18–75 years old and to be inclined to cooperate in the present study and exclusion criteria were having the kidney, liver, and lung diseases and other conditions that had negative effects on the cardiovascular or respiratory system health, or infectious and active inflammatory diseases, pregnancy, lactation, routine supplement or drug use, like weight loss, hormonal, sedative drugs, thermogenic supplements such as caffeine and green tea, conjugated linoleic acid (CLA), etc. The study protocols were approved by the ethical committee of Tehran University of Medical Sciences and in accordance with the Declaration of Helsinki. After informing subjects in detail about the study aims, written informed consent was obtained from all of them.

### Demographic data

Data on age, sex, education level, marital status, smoking, occupation, and the number of diseases was collected by demographic questionnaire.

### Physical activity

A validated International Physical Activity Questionnaire (IPAQ) was used to assess subjects’ physical activity levels. Recorded amounts were presented based on Metabolic Equivalents (METs) and categorized into three classes (very low: < 600, low: 600–3000, and moderate and high > 3000 MET-min/week) [15].

#### Anthropometric and blood pressure assessment

Weight was measured with light clothing and without shoes using a digital scale (808Seca, Germany) to the nearest of 0.1 kg and the height was estimated while standing and keeping the shoulders and hips against the wall without shoes, using a stadiometer (Seca, Germany) with an accuracy of 0.1 cm. Body mass index (BMI) was calculated as weight divided by squared height and presented as kg/m^2^. Waist circumference (WC) was measured between the lower rib and iliac crest, using a tape meter, according to standard guidelines. Waist to hip ratio (WHR) was calculated as waist circumference (cm) divided by hip circumference (cm).

After enough rest (at least 10–15 min), blood pressure was obtained by a digital barometer (BC 08, Beurer, Germany) in sitting position, and the mean of two measurements reported for each person.

### Biochemical assessments

First, a 10 mL venous blood sample was obtained from each subject after 7–10 h of fasting, then centrifuged for 20 min. Fasting blood glucose (FBG) was measured using a commercial kit (Pars Azmoon, Tehran) by enzymatic colorimetric assay (glucose oxidase). High-density lipoprotein (HDL-C) was assessed by the cholesterol oxidase phenol-amino-pyrine method, and triglyceride (TG) was measured by the enzymatic method of glycerol-3-phosphate oxidase phenol-amino-pyrene with automatic apparatus (Selecta E, Vitalab, Netherland).

### Dietary assessment

Usual dietary intake was estimated using a valid and reliable 168-item Food Frequency Questionnaire (FFQ) [16] which included a list of groceries and a standard size of each food item and was administered by skilled dietitians via face-to-face interviews. Participants were asked to report the frequency of consumption of each item on a daily, weekly, monthly, and annual basis.

Converting of consumed food portion sizes to grams was done by household measures [17] and calculated using a modified version of NUTRITIONIST IV software for Iranian foods (version 7.0; N-Squared Computing, Salem, OR, USA).

### Calculation of MIND diet score

We used dietary intakes obtained from FFQ to calculate the MIND diet score. This diet score was included 15 food items which were classified to brain-healthy and unhealthy food groups. The first food group contained 10 items such as green leafy vegetables, other vegetables, nuts, berries, beans, whole grains, fish, poultry, olive oil, and wine [18]. However, wine consumption was excluded. This beverage is consumed generally low in Muslim countries and prohibited in Islam, so the information on its consumption among Iranian is limited. Red meats, butter and stick margarine, cheese, pastries and sweets, and fast/fried food, are also considered as an unhealthy food group. To estimate the MIND diet score, we categorized participants based on tertile groups of the above-mentioned components’ intakes to minimize misclassification. Participants in the first, second, and third tertiles of brain-healthy food groups’ intake were given a score of 0, 0.5, and 1, respectively. Moreover, in brain-unhealthy food groups, we advocated scores of 1, 0.5, and 0 to individuals of the lowest, middle, and the highest tertiles, in order. Finally, the total MIND diet score was obtained by summing up the scores of these food items and ranged from 0 to 13 [19].

#### Obesity and MetS definition

General obesity was defined as BMI ≥ 30 kg/m^2^. Further, WC ≥ 102 cm for men and ≥ 88 cm for women, were considered as central obesity risk factors [20].

The presence of at least 3 of the following criteria was considered as MetS: (1) abdominal obesity (WC ≥ 102 cm for men and ≥ 88 cm for women); (2) low concentrations of HDL-C (< 50 mg/dL for women and < 40 mg/dL for men); (3) high serum TG levels (≥ 150 mg/dL); (4) abnormal homeostasis of glucose (FBS > 100 mg/dL); and (5) elevated blood pressure (systolic blood pressure ≥ 130 mmHg or diastolic blood pressure ≥ 85 mmHg) [21].

#### Data analysis

All statistical analyses were done using the Statistical Package for the Social Sciences (SPSS version 25; SPSS Inc.). We considered P < 0.05 as the significance level. The normality test was performed by the Kolmogorov–Smirnov test and also the Q–Q plot. We analyzed the study participants’ characteristics according to the MIND diet score tertiles, using one-way analysis of variance (ANOVA) and χ^2^ tests for continuous and categorical variables, respectively. Data are shown as the mean ± SD for continuous variables and percent (%) for categorical ones. Analysis of covariance (ANCOVA) was conducted to compare variables across the tertiles of the MIND diet score after controlling for confounders such as age, gender, marital status, physical activity, educations status, occupation, smoking, energy intake, and BMI. Odds ratio and 95% confidence intervals were obtained using logistic regression to determine the relationship of the MIND diet score with obesity and MetS. The risk was reported in crude and 3 adjusted models. In this analysis, the first tertile of exposure was considered as the reference category.

## Results

Mean age of study participants was 47.7 ± 10.7 years and 584 of them were female. MetS was prevalent among 36.1% (307) of study participants and mean BMI and WC in the whole study population was 27.7 ± 4.69 kg/m^2^ and 92.0 ± 12.4 cm, respectively. Demographic characteristics and anthropometric measures of participants across tertiles of the MIND diet score are shown in Table 1. There were no significant statistical differences in mean WC, BMI, SBP, and DBP and distribution of sex, smoking, education, occupation, metabolic disorders, marital status, and physical activity across tertiles of the MIND diet score. There were statistical differences in the distribution of general obesity (P = 0.01) and reduced serum HDL-C (P = 0.002).

**Table 1:** General characteristics of the participants in the study based on tertiles (T) of MIND diet score

	MIND diet score			P-value
	T1 (< 6) (n = 294)	T2 (6.5–7.5) (n = 278)	T3 (8<) (n = 264)	
	Mean ± SD			
Age (year)	43.6 ± 10.7	45.9 ± 10.6	44.7 ± 10.7	0.03
Weight (kg)	72.3 ± 12.7	74.1 ± 14.4	74.3 ± 13.4	0.16
BMI (kg/m^2^)	27.2 ± 4.22	28.1 ± 5.06	27.9 ± 4.69	0.09
WC (cm)	91.7 ± 11.8	93.0 ± 12.7	91.7 ± 12.4	0.34
Systolic blood pressure (mmHg)	119.8 ± 19.1	119.7 ± 24.4	119.3 ± 23.1	0.95
Diastolic blood pressure (mmHg)	78.4 ± 11.9	77.9 ± 14.7	78.3 ± 14.8	0.79
	N (%)			
Sex, n (%)				0.37
Male	83 (31.8)	93 (35.6)	85 (32.6)	
Female	211 (36.7)	185 (32.2)	179 (31.1)	
Smoking, n (%)				0.09
Not smoking	278 (36.6)	248 (32.7)	233 (30.7)	
Quit smoking	6 (18.2)	13 (39.4)	14 (42.4)	
Smoking	10 (22.7)	17 (38.6)	17 (38.6)	
Education, n (%)				0.43
Illiterate	23 (31.9)	28 (38.9)	21 (29.2)	
Under diploma	75 (33.8)	71 (32.0)	76 (34.2)	
Diploma	102 (39.8)	75 (29.3)	79 (30.9)	
Educated	94 (32.9)	104 (36.4)	88 (30.8)	
Occupation, n(%)				0.89
Employee	74 (34.3)	77 (35.6)	65 (30.1)	
Housekeeper	172 (36.4)	148 (31.4)	152 (32.2)	
Retired	40 (32.5)	43 (35.0)	40 (32.5)	
Unemployed	8 (32.0)	10 (40.0)	7 (28.0)	
Metabolic disorders, n (%)				0.36
Yes	184 (33.4)	187 (33.9)	180 (32.7)	
No	108 (38.3)	90 (31.9)	84 (29.8)	
Marital status, n (%)				0.87
Single	35 (39.3)	28 (31.5)	26 (29.2)	
Married	236 (34.9)	224 (33.1)	217 (32.1)	
Divorced	23 (32.9)	26 (37.1)	21 (30.0)	
Physical activity, n (%)				0.23
Low	177 (33.3)	175 (33.0)	179 (33.7)	
Moderate	117 (38.5)	102 (33.6)	85 (28.0)	
High	0 (0)	1 (100)	0 (0)	
General obesity	69 (29.1)	95 (40.1)	73 (30.8)	0.01
Components of metabolic syndrome				
Abdominal adiposity	137 (33.5)	148 (36.2)	124 (30.3)	0.21
Elevated blood pressure	103 (35.5)	100 (34.5)	87 (30.0)	0.73
High serum TG	112 (35.4)	102 (32.3)	102 (32.3)	0.88
Reduced serum HDL-C	148 (41.9)	106 (30.0)	99 (28.0)	0.002
Abnormal glucose homeostasis	138 (36.1)	117 (30.6)	127 (33.2)	0.32

Values are based on mean ± standard deviation or reported percentage

One-way ANOVA for quantitative data and Chi-2 test for qualitative data have been used

MIND diet score: Mediterranean-DASH Intervention for Neurodegenerative Delay; WC: Waist circumference; BMI: body mass index; mmHg: millimeter of mercury; kg: kilogram; kg/m^2^: kilogram per meter^2^

P value less than 0.05 was considered significant

Dietary intakes of macronutrients and components of MIND diet score across tertiles of the MIND diet score are indicated in Table 2. Participants in the highest tertile of the MIND diet score had greater intakes of green leafy vegetables, other vegetables, berries, beans, fish, poultry, and olive oil, and lower intakes of fast fried foods and pastries and sweets compared with those in the lowest tertile.

**Table 2:** Dietary intake of macronutrients and components of MIND diet score according to the tertiles (T) of the MIND diet score

	Tertiles of MIND diet score			P value	P for trend	P*
	T1 (< 6) (n = 294)	T2 (6.5–7.5) (n = 278)	T3 (8<) (n = 264)			
	Mean ± SD					
Energy, kcal/day	2504 ± 1168	2688 ± 3040	2506 ± 1199	0.46	0.99	–
Macronutrients						
Carbohydrates, g/day	371 ± 198	415 ± 759	366 ± 173	0.39	0.89	0.77
Protein, g/day	85.6 ± 45.2	88.9 ± 58.6	84.5 ± 38.1	0.53	0.79	0.87
Total fat, g/day	82.5 ± 45.3	80.9 ± 49.7	83.4 ± 59.1	0.84	0.83	0.40
Components of MIND diet score						
Brain healthy foods						
Green leafy vegetables, g/day	32.6 ± 26.0	54.9 ± 68.5	96.2 ± 88.4	< 0.001	< 0.001	< 0.001
Other vegetables, g/day	253 ± 139	380 ± 301	533 ± 352	< 0.001	< 0.001	< 0.001
Nuts, g/day	8.25 ± 34.1	8.07 ± 23.9	12.2 ± 31.6	0.19	0.11	0.19
Berries, g/day	3.80 ± 29.4	6.29 ± 23.3	14.2 ± 50.8	0.002	0.001	0.002
Beans, g/day	31.5 ± 42.8	40.4 ± 40.3	50.7 ± 50.7	< 0.001	< 0.001	< 0.001
Whole grains, g/day	1.64 ± 14.1	2.55 ± 14.7	4.01 ± 13.0	0.13	0.04	0.13
Fish, g/day	7.49 ± 10.5	11.6 ± 21.2	18.0 ± 29.0	< 0.001	< 0.001	< 0.001
Poultry, g/day	18.0 ± 28.6	24.8 ± 33.1	37.7 ± 46.5	< 0.001	< 0.001	< 0.001
Olive oil, g/day	0.40 ± 1.36	1.51 ± 4.58	2.61 ± 4.62	< 0.001	< 0.001	< 0.001
Brain unhealthy foods						
Cheese, g/day	25.0 ± 23.8	22.2 ± 30.1	20.6 ± 27.0	0.15	0.05	0.13
Red meat and products, g/day	43.5 ± 39.3	47.5 ± 55.7	49.5 ± 91.6	0.54	0.27	0.54
Butter and margarine, g/day	7.99 ± 12.7	6.66 ± 21.3	6.94 ± 22.0	0.67	0.51	0.68
Fast fried foods, g/day	21.1 ± 56.1	12.0 ± 22.0	10.2 ± 20.1	0.001	0.001	0.001
Pastries and sweets, g/day	81.0 ± 89.5	67.3 ± 87.4	66.8 ± 77.6	0.07	0.04	0.08

Values are based on mean ± standard deviation or reported percentage

One-way ANOVA have been used

Green leafy vegetables: cabbage, greens, lettuce, kale, spinach; other vegetables: green/red peppers, potato, peas or lima beans, tomatoes, tomato sauce, eggplant, onion, cucumber, squash, cooked carrots, raw carrots, broccoli, celery, corn, zucchini; berries: strawberries (strawberry, cherries, fresh berries)

Nuts: walnuts, pistachios, hazelnuts, almonds, peanuts; whole grains: dark bread (Iranian); fish: fish and tuna fish; beans: beans, lentils, peas, chick pea, soybeans; poultry: chicken, butter, margarine: butter, margarine, animal fats; cheese: cheese, red meat and products: red meat, hamburger, sausages; fast fried foods: French fries, pizza; pastries and sweets: biscuit, cake, chocolate, ice cream, confections, cocoa, Gaz (an Iranian confectionery made of sugar, nuts and tamarisk), cookies, candy, ice cream

*MIND diet score* Mediterranean-DASH Intervention for Neurodegenerative Delay

P value less than 0.05 was considered significant

*Adjusted for energy intake

The multivariate-adjusted means for TG, SBP, DBP, FBS, HDL-C, WC, and BMI according to the tertiles of the MIND diet score are shown in Table 3. In the crude model, there was no significant difference in terms of other components of MetS and BMI across tertiles of the MIND diet score. After controlling for covariates, these associations remained non-significant.

**Table 3:** The multivariate adjusted means for metabolic syndrome’s components and BMI according to tertiles (T) of MIND diet score

	MIND diet score			P-value	P_1_	P_2_
	T1 (< 6) (n = 294)	T2 (6.5–7.5) (n = 278)	T3 (8<) (n = 264)			
	Mean ± SD					
TG^ǂ^ (mg/dL)	147 ± 86.8	144 ± 77.8	144 ± 73.2	0.93	0.90	0.90
HDL^ǂ^ (mg/dL)	48.9 ± 10.4	50.5 ± 10.4	50.1 ± 9.71	0.13	0.20	0.20
FBS^ǂ^ (mg/dL)	107 ± 31.5	110 ± 61.2	106 ± 27.8	0.47	0.43	0.43
SBP^ǂ^ (mmHg)	119.8 ± 19.1	119.7 ± 24.4	119.3 ± 23.1	0.95	0.58	0.57
DBP^ǂ^ (mmHg)	78.4 ± 11.9	77.9 ± 14.7	78.3 ± 14.8	0.79	0.58	0.58
WC^ǂ^ (cm)	91.7 ± 11.8	93.0 ± 12.7	91.7 ± 12.4	0.34	0.25	0.24
BMI (kg/m^2^)	27.2 ± 4.22	28.1 ± 5.06	27.9 ± 4.69	0.09	0.29	0.33

Values are based on mean ± standard deviation

One-way ANOVA have been used

MIND diet score: Mediterranean-DASH Intervention for Neurodegenerative Delay; TG: triglyceride; HDL: high density lipoprotein; FBS: fasting blood sugar; SBP: systolic blood pressure; DBP: diastolic blood pressure WC: waist circumference; BMI: body mass index; mmHg, millimeter of mercury; kg: kilogram; kg/m^2^: kilogram per meter^2^

P value less than 0.05 was considered significant

P_1_: adjusted for age, gender, marital status, physical activity, educations status, occupation and smoking

P_2_: additionally, adjusted for energy intake

^ǂ^Also adjusted for BMI

Multivariate adjusted odds ratios and 95% CIs for MetS and its components across tertiles of the MIND diet score are provided in Table 4. In the crude model, although those who were in the third tertile of the MIND diet score compared to the first tertile were less likely to have MetS (OR = 0.88; 95% CI 0.62–1.24), there was no association between higher MIND diet score and MetS (P = 0.77). After adjusting for covariates, this result remained non-significant. Moreover, we found that adherence to the MIND diet was inversely associated with odds of reduced levels of HDL-C (OR: 0.59, 95% CI 0.42–0.83, P = 0.002) and general obesity (OR: 1.24, 95% CI 0.85–1.82, P = 0.01). When potential confounders were taken into account, such association remained significant for reduced levels of HDL-C (OR: 0.59, 95% CI 0.41–0.85, P = 0.008) and general obesity (OR: 1.19, 95% CI 0.80–1.78, P = 0.02). No significant association was seen between adherence to the MIND diet and abdominal obesity, elevated BP, elevated FBS, and increased serum TG in the crude and fully adjusted model.

**Table 4:** Odd ratios and 95% CIs for MetS and its components across tertiles of the MIND diet score

	MIND diet score			P value*
	T1 (< 6) (n = 294)	T2 (6.5–7.5) (n = 278)	T3 (8<) (n = 264)	
	OR (CI)			
MetS				
Crude	1	0.94 (0.67–1.32)	0.88 (0.62–1.24)	0.77
Model1	1	0.92 (0.64–1.33)	0.83 (0.57–1.21)	0.64
Model2	1	0.91 (0.63–1.31)	0.83 (0.57–1.20)	0.63
Model3	1	0.86 (0.55–1.16)	0.80 (0.55–1.16)	0.50
Reduced serum HDL				
Crude	1	0.60 (0.43–0.84)	0.59 (0.42–0.83)	0.002
Model1	1	0.62 (0.43–0.89)	0.59 (0.41–0.86)	0.008
Model2	1	0.62 (0.43–0.90)	0.59 (0.41–0.86)	0.009
Model3	1	0.62 (0.43–0.89)	0.59 (0.41–0.85)	0.008
Elevated BP				
Crude	1	1.04 (0.74–1.47)	0.91 (0.64–1.29)	0.73
Model1	1	0.85 (0.57–1.26)	0.79 (0.53–1.19)	0.51
Model2	1	0.83 (0.56–1.24)	0.79 (0.53–1.18)	0.49
Model3	1	0.79 (0.53–1.18)	0.76 (0.51–1.15)	0.37
Elevated FBS				
Crude	1	0.82 (0.59–1.14)	1.04 (0.75–1.46)	0.32
Model1	1	0.80 (0.56–1.13)	0.98 (0.69–1.38)	0.40
Model2	1	0.80 (0.56–1.13)	0.98 (0.69–1.38)	0.40
Model3	1	0.79 (0.56–1.12)	0.97 (0.68–1.37)	0.37
Increased serum TG				
Crude	1	0.94 (0.67–1.32)	1.02 (0.72–1.44)	0.88
Model1	1	0.97 (0.69–1.38)	1.02 (0.72–1.44)	0.97
Model2	1	0.96 (0.68–1.36)	1.01 (0.71–1.44)	0.95
Model3	1	0.97 (0.68–1.37)	1.02 (0.72–1.45)	0.96
Abdominal obesity				
Crude	1	1.30 (0.93–1.81)	1.01 (0.72–1.41)	0.21
Model1	1	1.29 (0.88–1.88)	0.93 (0.64–1.36)	0.22
Model2	1	1.27 (0.87–1.86)	0.93 (0.63–1.36)	0.25
Model3	1	1.17 (0.78–1.76)	0.84 (0.56–1.26)	0.29
General obesity				
Crude	1	1.69 (1.17–2.44)	1.24 (0.85–1.82)	0.01
Model1	1	1.71 (1.16–2.52)	1.20 (0.80–1.79)	0.02
Model2	1	1.69 (1.14–2.49)	1.19 (0.80–1.78)	0.02

Data are presented as odds ratio (95% CI)

Model 1: adjusted for age, gender, marital status, physical activity, educations status, occupation and smoking

Model 2: additionally, adjusted for energy intake

Model 3: further adjustment was made for BMI

*MIND diet score* Mediterranean-DASH Intervention for Neurodegenerative Delay, *MetS* metabolic syndrome, *WC* waist circumference, *FBS* fasting blood glucose, *TG* triglyceride, *HDL* high-density lipoprotein, *SBP* systolic blood pressure, *DBP* diastolic blood pressure

*Obtained by binary logistic regression

## Discussion

In the present study, we found a non-significant inverse association between adherence to the MIND diet and odds of MetS and abdominal obesity. However, our findings showed a negative significant relationship between the MIND diet score and odds of reduced levels of HDL-C and general obesity. Such significant association was also seen even after taking potential confounders in to account. To the best of our knowledge, this is the first study that examined the relationship between the MIND diet score with MetS and its components and general and abdominal obesity in Iranian adult population.

Generally, the MIND dietary pattern is a combination of the MD and the DASH diet which differs by assigning separate groups for green leafy vegetables and berries, as well as cakes and pastries. In comparison to the MD and DASH diet, fruit was omitted and fish was not administered regularly, because according to some evidence 2–3 times a week is appropriate for neuroprotective effects [11]. Several studies have assessed the link between dietary pattern and MetS [7, 9]; however, little attention has been paid on the MIND diet. In the current study, we observed a non-significant association between adherence to the MIND diet and the odds of Mets.

Although there are no observational studies that directly assessed the association between adherence to the MIND diet and MetS, several documents have addressed the linkage between the DASH diet and MD and MetS. In agreement with our findings, some studies have shown no significant association between DASH [7] or MD [10] and MetS. Soric et al. [7] in sixty-seven hospitalized schizophrenic patients did not see a significant association between the DASH diet and the prevalence of MetS and its components. In addition, a cross-sectional study on 157 T2D patients, did not show an association between MD and MetS [10]. In contrast, Ghorabi et al. [6] in a sample of 396 Iranian adults, found a significant inverse association between adherence to the DASH diet and odds of MetS, but in line with our study, they found that adherence to DASH diet was inversely associated with reduced levels of HDL-C. Also, a systematic review indicated the beneficial effect of adherence to the MD on the incidence and development of MetS [22].

Another important finding of this study was the inverse significant association between the MIND diet and odds of low serum HDL-C, but no significant association was observed with other components of MetS. In accordance with our finding, Azadbakht et al. [23] in an 8-week randomized trial in 31 patients with T2D suggested the DASH diet could increase HDL-C. In contrast, in the study of Obarzanek et al. [24] DASH diet resulted in lower HDL-C, which can be explained by lower total dietary fat intake.

In this study, we found a significant decrease in general obesity and a non-significant decrease in abdominal obesity following adherence to the MIND diet score. There is limited evidence in this area. In line with our results, Esposito et al. [25] in a meta-analysis, suggested a beneficial effect of MD on weight regardless of energy intake, and also, they declared that energy restriction increased the weight loss caused by a MD. Another meta-analysis showed that adherence to the DASH diet significantly decreases body weight (about—1.42 kg in 8–24 weeks), BMI, and WC, especially along with energy-restricted diets [26]. In contrast, Aminianfar et al. [27] found no significant association between adherence to the MIND diet and odds of general and central obesity in both men and women. However, they did not include olive oil in the score and their sample size was lower.

The discrepancies between these studies and the results of this study can be explained by different amounts of fiber, potassium, and ca. in DASH, MD, and the MIND diet. In the MIND diet dairy is limited to just cheese and fruits are limited to berries. Studies have shown that dairy products are inversely related to the MetS, body weight, and insulin resistance therefore might have beneficial effects on all metabolic disorder characteristics [28]. Calcium’s beneficial effect on the prevention of fat accumulation can also be due to the expression of the uncoupled protein (UCP2) in the white adipose tissue and help thermogenesis and reduce waist circumference [29]. Additionally, casein and conjugated linolenic acid may also play a role in preventing the accumulation of fat [30]. Lower weight and waist circumference can ultimately contribute to lower blood pressure [31].

Fruits are good sources of fiber, antioxidants, and phytochemicals, which can play an important role in weight control [32], suppressing reactive oxygen species and delaying the progression of systemic oxidative damage [33], regulating inflammatory markers [34], and prevention of insulin resistance and MetS [35]. Fruits and vegetables also have sufficient amounts of potassium. Interventional studies have suggested a protective effect of potassium on blood pressure and hypertension [36]. However, Vendrame et al. [37] in their review suggested that regular intake of berries as part of a healthy diet is a promising strategy to prevent MetS and its complications. Oxidative stress is a common characteristic of MetS [38]. Berries are good sources of polyphenols (including flavonoids), tannins, phenolic acids, and lignans [39]. Ruel et al. administered three different doses (125, 250, and 500 mL/day) of cranberry juice in 30 middle-aged men with abdominal obesity for 4 weeks. They saw a significant decrease in body weight, BMI, waist circumference, total/HDL-C ratio, HDL-C and apolipoprotein B, and a significant increase in plasma total antioxidant capacity after 250 mL and/or 500 mL consumption [40].

The beneficial effects of the MIND diet may have been linked to the use of olive oil as the main source of dietary fats and phenolic compounds. George et al. [41] reported that high polyphenol olive oil can improve total and HDL-C and related parameters of oxidative stress. Tsartsou et al. [42] indicated that the major effect of olive oil high in polyphenols is the increase of HDL-C circulation. Also, some randomized controlled trials showed improvement in endothelial function and insulin sensitivity and secretion by olive oil phenolic compounds and oleic acid [43–45].

Reduction in general obesity in this study may be due to many components of the MIND diet. For example intake of less high-calorie-dense foods in the third tertile of the MIND diet score [46]. Also, this diet contains plant-based and low-glycemic index foods that have high dietary fiber, water content, and low glycemic load [47]. Therefore promotes weight loss [48] and negatively influences MetS features [49] such as regulating blood glucose [50], and lipid profile [49].

Our study had some strengths, to the best of our knowledge, this is the first study to investigate the association between the MIND diet score and MetS and general and abdominal obesity. We adjusted for several known factors that may influence the results also the analysis was conducted on a large sample with a wide age range of adults in a Middle East population. In addition, a validated FFQ which provides accurate and reliable information was used to assess dietary intakes. However, it should be mentioned that this study had some limitations because of the cross-sectional design, and no causal associations can be identified, the residual confounders cannot be removed whilst we have controlled for possible confounding. Further well-designed and long term studies with larger sample sizes are needed to confirm these findings.

## Conclusions

In conclusion, significant associations were found between adherence to the MIND diet and odds of reduced levels of HDL-C and general obesity. Given the importance of chronic diseases such as MetS and obesity in health status, further investigations are needed.

## Data Availability

Since the privacy of research participants may be compromised, we cannot make the information publicly available.

## Abbreviations

MIND: Mediterranean-DASH Intervention for Neurodegenerative Delay
MetS: Metabolic syndrome
FFQ: Food frequency questionnaire
ATP III: Adult Treatment Panel III
BMI: Body mass index
WC: Waist circumference
OR: Odds ratio
HDL-C: High-density lipoprotein cholesterol
CVDs: Cardiovascular diseases
T2D: Type 2 diabetes
FBS: Fasting blood sugar
NCEP: National Cholesterol Education Program
MD: Mediterranean dietary pattern
DASH: Dietary Approaches to Stop Hypertension
TG: Triglycerides
CLA: Conjugated linoleic acid
IPAQ: Physical Activity Questionnaire
METs: Metabolic Equivalents
WHR: Waist to hip ratio
FBG: Fasting blood glucose
UCP2: Uncoupled protein
